# Phytochemical Profile and Biological Activities of Tendrils and Leaves Extracts from a Variety of *Vitis vinifera* L.

**DOI:** 10.3390/antiox9050373

**Published:** 2020-04-30

**Authors:** Mirela L. Moldovan, Rahela Carpa, Ionel Fizeșan, Laurian Vlase, Cătălina Bogdan, Sonia M. Iurian, Daniela Benedec, Anca Pop

**Affiliations:** 1 Department of Dermopharmacy and Cosmetics, Faculty of Pharmacy, “Iuliu Hațieganu” University of Medicine and Pharmacy, 12 I. Creangă Street, 400010 Cluj-Napoca, Romania; mmoldovan@umfcluj.ro (M.L.M.); catalina.bogdan@umfcluj.ro (C.B.); 2 Department of Molecular Biology and Biotechnology, Faculty of Biology and Geology, “Babeș-Bolyai” University, 1 M. Kogălniceanu Street, 400084 Cluj-Napoca, Romania; rahela.carpa@ubbcluj.ro; 3 Department of Toxicology, Faculty of Pharmacy, “Iuliu Hațieganu” University of Medicine and Pharmacy, 6 L. Pasteur Street, 400349 Cluj-Napoca, Romania; ionel.fizesan@umfcluj.ro (I.F.); anca.pop@umfcluj.ro (A.P.); 4 Department of Pharmaceutical Technology and Biopharmacy, Faculty of Pharmacy, “Iuliu Hațieganu” University of Medicine and Pharmacy, 41 V. Babeș Street, 400012 Cluj-Napoca, Romania; laurian.vlase@umfcluj.ro (L.V.); sonia.iurian@umfcluj.ro (S.M.I.); 5 Department of Pharmacognosy, Faculty of Pharmacy, “Iuliu Hațieganu” University of Medicine and Pharmacy, 12 I. Creangă Street, 400010 Cluj-Napoca, Romania

**Keywords:** tendrils, extract, cytocompatibility, antioxidant, anti-inflammatory, antimicrobial, *Vitis vinifera*

## Abstract

Winery industry by-products have a great reuse potential in the pharmaceutical and cosmetic fields due to their bioactive compounds. This study investigates the phytochemical profile and the bioactivity of *Vitis vinifera* variety Fetească neagră tendrils extract (TE) and leaves extract (LE), intended to be used in oral hygiene products recommended in periodontal disease. The evaluation of the phenolic content was performed by colorimetric analysis. Liquid chromatography coupled with mass spectrometry was used to determine the chemical profile of the two extracts obtained from *V. vinifera*. Moreover, the antioxidant activity of the extracts was determined by spectrophotometric methods, as well as on human gingival fibroblasts (HGF) cell line. The cytocompatibility and cytoprotective effect against nicotine-induced cytotoxicity were tested, as well as the anti-inflammatory and antimicrobial effects. The TE showed higher total polyphenolic content, rich in rutin, compared to the leaves extract that displayed important amounts of isoquercitrin. The antioxidant effect was confirmed by both non-cellular and cellular tests. The cytocompatibility of the extracts was confirmed at a wide range of concentrations. The cytoprotective effect was demonstrated in HGF exposed to cytotoxic doses of nicotine; 300 µg/mL of both tested extracts decreased nicotine toxicity by approximately 20%. When challenged with *E. coli* polysaccharides, in HGF cells co-exposed to TE and LE, a reduction in the release of proinflammatory cytokines (IL-8, IL-6 and IL-1β) was observed. The extracts were both able to reduce the levels of reactive oxygen species and inflammatory cytokines, and had notable antimicrobial effects on pathogenic bacteria associated with periodontitis.

## 1. Introduction

*Vitis vinifera* L. (*V. vinifera*) is a plant that belongs to Vitaceae family, which has been cultivated for more than 7500 years and is currently considered one of the most important fruit crops due to the great surfaces cultivated worldwide [1,2]. The global production rises to more than 67 million tons per year, among which eighty percent are processed for winemaking [3]. The winery industry is known for generating important amounts of by-products, the disposal of which can pose economic and environmental problems as they account for about 30% of the processed grapes [4]. The food wastage problem has attracted high public attention and led the European Parliament to adopt a resolution that envisages strategies to diminish the loss in the food chain [5]. In this context, researchers and companies have tested several solutions for winery by-product reuse, such as biogas production out of the semisolid residues [4], animal feed or soil amendments out of grape stems, and extraction of grape seed oil and polyphenols out of grape pomace [6].

A significant number of scientific papers regarding grape processing by-products have been published so far, mainly focusing on pomace or on grapes’ seeds, stem, and skin, but a limited number of studies have been reported on leaves and tendrils of *V. vinifera*. The scarce information available with regard to grape leaves indicates the presence of organic acids, phenolic acids, flavonols, tannins, procyanidins, anthocyanins, enzymes, vitamins, and carotenoids, and therefore a high nutritional and biological potential [6]. One of the few studies focused on tendrils’ composition reveals flavonoids, polyphenols, and anthocyanins as major constituents with promising in vitro anti-inflammatory effects [7]. Due to the rich content in bioactive compounds of winery by-products, the research conducted so far was focused on the mechanisms of actions of these phytochemicals and their possible use as food supplements; however, less attention was paid to their possible beneficial effects on oral health and disease prevention and progression at this level.

In this context, the present study aimed to characterize the phytochemical composition of the less studied extracts of leaves and tendrils from *V. vinifera* subsp. *vinifera* cultivated variety (c.v) Fetească neagră (FN) and, for the first time, their applicability in the management of periodontal disease. The tendrils extract (TE) and leaves extract (LE) were characterized by quantifying the total polyphenolic, flavonoid, and acid caffeic derivatives content by colorimetric methods and subsequently by liquid chromatography coupled with mass spectrometry in tandem (LC-MS/MS) methods. The antioxidant activity of the extracts was investigated through in vitro non-cellular assays (FRAP and DPPH methods), considering the complex role of reactive oxygen species in the pathophysiology of periodontal disease. The potential application of polyphenols in periodontal disease is mainly due to their effects on inflammation signals, antioxidant and antibacterial activity. Polyphenols, such as epigallocatechin, quercetin, and caffeic acid, have already been confirmed for their in vitro cytoprotective action on the cells exposed to nicotine or lipopolysaccharides [8,9,10].

As elements of novelty, the applicability of TE and LE in the management of periodontal disease was investigated by studying the antioxidant and anti-inflammatory potentials in human gingival fibroblast cell culture, as oxidative stress imbalance and inflammatory processes underlay the pathophysiological alterations noted in periodontal disease; the cytoprotective effect of the extracts against nicotine was also investigated: nicotine being responsible for the production of free radicals and the oxidative stress, with consequences on gingival and periodontal ligament fibroblast functions [11].

Furthermore, the antimicrobial activity of these extracts was tested on several bacterial strains associated with the host inflammatory processes noted in periodontal disease.

## 2. Materials and Methods

### 2.1. Preparation of Leaves and Tendrils Extract

#### 2.1.1. Plant Material

The leaves and tendrils of *V. vinifera* subsp. *vinifera* cultivated variety (c.v.) Fetească neagră were harvested in July 2019 from the experimental fields of the Research Centre for Viticulture and Oenology Murfatlar, Romania (44°10′49,73′′N; 28°25′28,67′′E). A voucher specimen is deposited in the herbarium of the SCDVV Murfatlar Constanta County (Voucher No. 55). The plant materials were dried in Excalibur Dehydrator (4500220FB) at 30 °C for 24–48 h and then ground in a grinder (Zass ZCG 07) to a fine powder and sieved through a 1 mm sieve.

#### 2.1.2. Preparation of Extracts

The extracts from *V*. *vinifera* leaves and tendrils were obtained by reflux method, on water bath, for 30 min at 80 °C with 50% ethanol (*v/v*). To obtain the extracts, 10 g of plant material powder and 90 mL of 50% ethanol were used. Then, after cooling, the plant residue was separated by filtration through fluted filter paper. To remove the insoluble materials, the extracts were centrifuged (1930× *g*) for 20 min, and the supernatants were recovered [12,13].

### 2.2. Quantitative Determination of Bioactive Compounds

#### 2.2.1. Chemicals

The standard substances used for LC-MS analysis and spectrophotometric methods were purchased from the following specialized companies. Sigma, St. Louis, MO, USA (chlorogenic acid, p-coumaric acid, caffeic acid, rutin, apigenin, quercetin, isoquercitrin, quercitrin, hyperoside, kaempferol, myricetin, and fisetin); Roth Karlsruhe, Germany (ferulic acid, sinapic acid, gentisic acid, gallic acid, patuletin, and luteolin); Dalton Toronto, Canada (cichoric acid, caftaric acid); Sigma-Aldrich, Steinheim, Germany (protocatechuic, vanillic and syringic acids, as well (–)-epicatechin and (+)-catechin). The reagents and chemicals used were ethanol, methanol, sodium acetate, aluminum chloride, Arnow’s reagent (sodium nitrite, sodium molybdate), hydrochloric acid, Folin-Ciocâlteu reagent, 2,2-diphenyl-1-picrylhydrazyl (free radical, DPPH), HPLC (High Performance Liquid Chromatography)-grade methanol, and acetic acid were purchased from Merck, Darmstadt, Germany. 2,4,6-Tris(2-pyridyl)-s-triazine (TPTZ), 6-hydroxy-2,5,7,8-tetramethylchroman-2-carboxylic acid (Trolox), and sodium acetate buffer solution were acquired from Sigma-Aldrich, Steinheim, Germany. Sodium hydroxide, sodium carbonate, and iron (III) chloride (ferric chloride) were obtained from Alfa-Aesar, Karlsruhe, Germany. All chemicals and reagents were of high-grade purity. The chemicals used to evaluate the antioxidant activity on cell cultures were dimethyl sulfoxide (DMSO) (≥99%), hydrogen peroxide 30% solution, N-acetyl-l-cysteine (≥99%), phosphate buffer, resazurin, 2,7 dichloro-fluorescein diacetate (DCFH-DA), lipopolysaccharides from *E. coli*, acquired from Sigma Aldrich (Schnelldorf, Germany). Thermo Fisher Scientific (Gibco, Paisley, UK) was the supplier for the Dulbecco’s modified Eagle’s medium (DMEM), and Sigma Aldrich (Stenheim, Germany) for the fetal bovine serum (FBS). ELISA kits for the quantification of IL-6 and IL-8 were acquired from Biolegend (San Diego, CA, USA), while the IL-1β kit was obtained from Biogems (Westlake Village, CA, USA). For antimicrobial activity testing Nutrient agar (Oxoid, UK), Sabouraud Dextrose agar (Oxoid, UK) and Müeller–Hinton agar (Oxoid, UK) were used.

#### 2.2.2. Determination of Total Flavonoid and Caffeic Acid Derivatives Content

Total flavonoid contents (TFC) from the *V*. *vinifera* LE and TE were evaluated by flavonoid–aluminum chloride (AlCl_3_) complexation method described in the Romanian Pharmacopoeia X^th^ edition [14]. To 5 mL of each extract, 5.0 mL 10% sodium acetate solution and 3.0 mL 2.5% aluminum chloride solution were added and filled up to 25 mL with methanol in a calibrated flask. The absorbance of each sample was measured after a reaction time of 15 min, using a Jasco model V-530 spectrophotometer (Jasco International Co., Ltd., Tokyo, Japan) set at 430 nm. A blank solution was similarly prepared but adding methanol instead of aluminum chloride. The absorbance was measured at 430 nm after a reaction time of 15 min. Rutin was used as the standard reagent to obtain the calibration curve (*R*^2^ = 0.999). The results were expressed as mg rutin equivalents RE/g dry weight (d.w.) [14,15]. All the samples were analyzed in triplicate.

A spectrophotometric method was used to determine the contents of caffeic acid derivatives in the two samples, according to the method described in Romanian Pharmacopoeia X^th^ edition, using Arnow’s reagent (10 g sodium nitrite, 10 g sodium molybdate, and distilled water up to 100 mL) [14]. The percentage of caffeic acid derivatives, expressed as caffeic acid equivalents (CAE) in dry product (mg CAE/g d.w.), was determined from the equation of the calibration curve. A standard curve was obtained using different concentrations of caffeic acid standard (*R*^2^ = 0.994) [14,16]. The samples were analyzed in triplicate.

#### 2.2.3. Determination of Total Phenolic Content

The total polyphenol content (TPC) in the *V*. *vinifera* extracts was measured spectrophotometrically according to the Folin-Ciocalteu method [17,18]. Gallic acid was used as standard phenolic compound. Briefly, 1.0 mL Folin-Ciocalteu reagent, 10.0 mL distilled water, and 29% sodium carbonate solution were added to 0.5 mL extract in a 25 mL graduated flask. After 30 min of incubation in the dark, the absorbance of the mixture was measured at 760 nm using distilled water as compensation liquid. TPC expressed as mg gallic acid equivalents (GAE)/g of dry plant material was obtained from a previously developed calibration equation (*R*^2^ = 0.998) [13,15,16].

All the samples were analyzed in triplicate.

#### 2.2.4. Phytochemical Analysis by LC-MS/MS

The phytochemical analysis of the extracts was performed by liquid chromatography coupled with mass spectrometry in tandem (LC-MS/MS) on an Agilent 1100 HPLC Series system (Agilent, Santa Clara, CA, USA) equipped with degasser, autosampler, binary gradient pump, column thermostat, and UV detector. The HPLC system was coupled with an Agilent Ion Trap 1100 SL mass spectrometer (LC/MSD Ion Trap VL).

A reverse-phase analytical column (Zorbax SB-C18, 100 mm × 3.0 mm i.d., 3.5 µm) was used for separation, with a mobile phase consisting in a mixture of methanol: 0.1% acetic acid (*v/v*) and a binary gradient (first a linear gradient from 5% methanol to 42% methanol for 35 min; isocratic elution, then 42% methanol for the next 3 min, followed by rebalancing with 5% methanol over the next 7 min). The flow rate was 1 mL/min, the injection volume was 5 µL and the column temperature 48 °C with combined detection: UV (330 nm, 370 nm) and MS mode. ChemStation and DataAnalysis software from Agilent, Santa Clara, CA, USA were used to process the chromatographic data.

The same analytical conditions were used to detect catechin, epicatechin, gallic acid, syringic acid, vanillic acid, and protocatechuic acid, but using a different binary gradient (start with 3% methanol; over 3 min 8% methanol, from 8.5 min until 10 min with 20% methanol, then 3% methanol to rebalance column) and compounds detection in MS mode.

In both cases, the MS system operated using an electrospray ion source in negative mode (capillary +3000 V, nebulizer 60 psi (nitrogen), dry gas nitrogen at 12 L/min, dry gas temperature 360 °C) [19].

The identified compounds were quantified based on their peak area and the calibration curve of their corresponding standards and the results were expressed as µg of polyphenolic compound/g dry product.

### 2.3. Antioxidant Activity Evaluation

To measure the antioxidant activity of the *V. vinifera* extracts, two commonly used methods were chosen: DPPH and FRAP assays. Moreover, it is known that wine polyphenols have an antioxidant effect that is based on the ability to supply the hydrogen atom from their hydroxyl groups [15,16,20].

#### 2.3.1. DPPH Radical Scavenging Activity

The antioxidant potential of the *V*. *vinifera* LE and TE was assessed according to the previously described DPPH method [12,14,16]. Briefly, a DPPH radical solution (0.1 g/L) in methanol was prepared and 2.0 mL of this solution was added to 2.0 mL of extract solution (or standard) in methanol at different concentrations (0.0625–0.3125 mg/mL). The absorbance of the samples (As) and the control solutions (Ac—absorbance of DPPH radical + methanol, containing all reagents except the sample) were measured at 517 nm, after half an hour. The decrease in the absorbance was measured at 517 nm. The antiradical activity (three replicates per treatment) was expressed as IC_50_ (μg/mL): the concentration of vegetal material required to cause a 50% DPPH inhibition [13,16].

#### 2.3.2. Ferric-Reducing Antioxidant Power (FRAP) Assay

The Ferric-Reducing Antioxidant Power (FRAP) assay evaluated the reduction of Fe^3+^-TPTZ (green) to blue-colored Fe^2+^-TPTZ complex by measuring the absorbance at 593 nm [21,22]. The FRAP reagent consists of a mixture of 2.5 mL 10 mM TPTZ solution in 40 mM HCl to which 2.5 mL of 20 mM ferric chloride (Fe^3+^) solution and 25 mL of acetate buffer (pH = 3.6) were added. A volume of 0.4 mL of diluted sample was further incubated with 6 mL of FRAP reagent and the absorbance of the reaction mixture was measured at 450 nm. The results were expressed as μmol Trolox equivalent/mL extract based on a standard curve (*R*^2^ = 0.992) using different concentrations of Trolox (10–40 mg/L) [21,23]. Analyses were performed in triplicate on each extract.

### 2.4. Biological Activities on Cell Lines

#### 2.4.1. Cell Culture

Normal human gingival fibroblasts (HGF) (CLS Cell Lines Service, Eppelheim, Germany) were maintained in DMEM supplemented with 10% FBS. Cells were cultured in flasks at 37 °C in a humidified incubator with 5% CO_2_ supplementation and the medium was changed every 2–3 days. The cells were used for experiments or subcultured once they reached 70–80% confluence.

#### 2.4.2. Cytocompatibility and Cytoprotective Effect against Nicotine-Induced Cytotoxicity

The cytocompatibility of the LE and TE was measured by Alamar Blue (AB) assay using the previously described protocol [24]. Briefly, the cells left to attach for 24 h were exposed to varying concentrations of LE and TE (10–400 μg/mL) for another 24 h. Following the exposure, the medium was carefully removed; the cells were washed with phosphate buffer saline (PBS) and incubated with a resazurin solution of 200 µM for 4 h. Viable cells have the ability to reduce resazurin to resorufin, a compound with intrinsic fluorescence, which was measured at λ_excitation_ = 530/25; λ_emission_ = 590/35 using Synergy 2 Multi-Mode Microplate Reader (BioTek Instruments, Winooski, VT, USA). Three biological replicates including each six technical replicates were performed and included a negative control (cells exposed to culture medium containing 0.2% DMSO). The results were represented as relative values compared to the negative control (100%).

The cytoprotective effect of the TE and LE against nicotine toxicity was evaluated using AB assay described above. HGF cells were exposed for 24 h to a mixture of the extracts (LE and TE) at three concentrations, 100, 200, and 300 µg/mL, and 10 µM nicotine (IC_50_) followed by the incubation with a resazurin solution of 200 µM for 4 h. Three biological replicates including each 6 technical replicates were performed and included a negative control (cells exposed to culture medium containing 0.2% DMSO). The results were represented as relative values compared to the negative control (100%).

#### 2.4.3. Antioxidant Activity on Cell Cultures

The ability of the LE and TE to mitigate the oxidative stress in HGF cells was evaluated using the ROS-sensitive dye 2ʹ,7ʹ-dichlorofluorescin diacetate (DCFH-DA) as previously described [25]. Briefly, after an incubation of 24 h to non-toxic concentrations of LE and TE (100, 200, and 300 µg/mL), the cells washed with PBS were further exposed for 2 h to 50 µM DCFH-DA in Hanks’ Balanced Salt Solution (HBSS). To measure the quantity of ROS in stimulated and non-stimulated conditions, the cells were exposed to 250 µM H_2_O_2_ or HBSS for 2 h, after removing the excess of DCFH-DA. The fluorescence of dichlorofluorescein (DCF) was measured using Synergy 2 Multi-Mode Microplate Reader at λ_excitation_ = 485/20; λ_emission_ = 528/20. The antioxidant properties of the LE and TE were compared to the N-Acetyl Cysteine (NAC) (20 mM solution).

#### 2.4.4. Anti-inflammatory Potential

The LE and TE anti-inflammatory potential was evaluated by measuring the levels of three proinflammatory cytokines, namely, IL-8, IL-6 and IL-1β, in cell culture supernatant using ELISA assays. HGF cells were concomitantly exposed to 100 ng/mL Lipopolysaccharides (LPS) from *E. coli* and to three non-cytotoxic concentrations of the LE and TE (100, 200, and 300 µg/mL) for 24 h. To exclude an additive cytotoxic effect of the extracts in the presence of LPS, the effect of LPS-extract mixtures on HGF viability was evaluated. Following the exposure, cell culture supernatants were removed and stored at −80 °C until analysis. The concentrations of IL-8, IL-6 and IL-1β were measured using commercially available ELISA Kits. A cytokine standard curve was included in each experiment, and cytokine levels were calculated from a four-parameter logistic curve according to the manufacturer’s instructions.

### 2.5. Antimicrobial Potential of the Extracts

A qualitative diffusimetric method adapted from disk/well method was used to test the microbiological activity of LE and TE. The method is based on the property of substances to diffuse into the nutrient environment, creating a circular area where concentrations of antibacterial substances are decreasing from center to periphery. In this study, the method of applying a volume of the test samples (120 μL) in 5 mm wells made in the culture medium was used. The samples consisted of the LE and TE obtained by the method presented above. All samples were inoculated on round, sterile swabs placed in the wells of the solid culture medium which was previously inoculated with a known bacterial culture. The test sample (LE and TE) diffuses into the environment around the disk. If the studied species is sensitive to the applied substance, it will not develop bacteria around the disk, the environment remaining clear, unlike the rest of the environment in the Petri dish, on which a canvas culture of the respective bacteria develops [26].

#### 2.5.1. Test Microorganisms

The microorganisms used in this test were *Streptococcus mutans* ATCC 25175, *Porphyromonas gingivalis* ATCC 33277, *Enterococcus faecalis* ATCC 29212, *Escherichia coli* ATCC 25922, *Staphylococcus aureus* ATCC 25923, *Klebsiella* sp., and *Candida albicans* ATCC 10231 (ATCC—American Type Culture Collection, Manassas, VA, USA).

#### 2.5.2. Culture Media

Nutrient agar, Sabouraud Dextrose agar, and Müeller-Hinton agar were used to grow the bacterial and fungal strains in Petri dishes. These culture media were prepared according to the manufacturer’s instructions.

#### 2.5.3. Method

Experiments were performed in a vertical laminar flow air hood Steril Helios (Bionova, Italy). Each bacterial strain was grown for 24 h on nutrient agar medium, and the fungal strains on Sabouraud agar culture medium [27]. Then, from each strain, a dilution of 0.5 McFarland in sterile physiological serum (NaCl 0.85%) was made. From these dilutions, each Petri dish is inoculated with a sterile swab soaked in the 0.5 McFarland microbial suspension and spread over the entire surface of the solid culture medium (Müeller-Hinton Agar (Oxoid, UK)). The Petri dishes were dried for 30 min at 37 °C. Afterwards, 5 mm diameter wells were carved in the agar using a cut sterile pipette tip. The wells were then filled with sterile cotton beads. Each bead was loaded with 120 µL of each extract (noted 1, 2, 3, and 4) and one control labeled C (ethyl alcohol/H_2_O 50/50 (*v/v*)). Incubation was done for 18–24 h and maximum 48 h at 37 °C (Salvis Incuccenter incubator IC400, SalvisLab, Switzerland). The reading was done by measuring the diameter of the inhibition zone: the larger the diameter of the inhibition zone, the greater the sensitivity of the bacterium to the respective antibacterial substances [26].

### 2.6. Statistical Analysis

All results are expressed as means ± standard deviation (SD). The data were statistically analyzed by one-way and two-way analysis of variance (ANOVA) (with post hoc Holm-Sidak for comparing multiple treatments) using Microsoft Office Excel 2016 (Microsoft Corporation, Redmond, WA, USA) and SigmaPlot 11.0 computer software (Systat Software, San Jose, CA, USA). The difference showing a *p* level of 0.05 or lower was considered statistically significant.

## 3. Results and Discussion

### 3.1. Total Phenolic Contents and Antioxidant Activity

The results obtained by spectrophotometric determinations for the two extracts of *V*. *vinifera* LE and TE are presented in Table 1.

The highest amount of the total flavonoids (TFC) and phenolic acids was determined in the extract of *V*. *vinifera* LE (16.75 mg/g TFC and 6.39 mg/g caffeic acid derivatives, respectively) followed by *V*. *vinifera* TE (14.21 mg/g and 4.14 mg/g, respectively). Concerning the content of total polyphenol (TPC), however, the TE (35.65 mg/g) was richer in polyphenolic compounds than the LE (28.62 mg/g). The values obtained (Table 1) suggest that highly significant differences were revealed between the two extracts (LE versus TE) in terms of flavonoid, caffeic acid derivatives, and total polyphenolic amounts (*p* < 0.001).

Regarding the phenolic content of the leaves, due to the type of extract, the pedo-climatic conditions, the variety, the harvesting period, etc., our results were either similar [28,29], or different from the results published by other authors [28,29,30,31,32,33]. As for the total flavonoid content, the extract of *V*. *vinifera* leaves from Romania was richer than some grape varieties from Turkey, Croatia, or India [28,31,34]. In contrast, the same plant product had a lower concentration of flavonoids than those harvested from species growing in Anatolia or Hungary [32,35]. Data on the total content of caffeic acid derivatives from the leaves are scarce.

By comparison with our values of TPC in grape leaves, those reported by others authors on the grapevine leaves from Algeria or Montenegro revealed higher amounts of TPC [28,29,30], whereas some varieties from Turkey, Portugal, and Croatia displayed lower concentrations than ours [28,31,32,33].

The evaluation of antioxidant activity of the extracts by DDPH method showed that the *V. vinifera* TE (0.155 mg/mL) presented a significantly greater effect than *V. vinifera* LE (0.248 mg/mL) (*p* < 0.05). The lower IC_50_ values show a good antioxidant capacity. IC_50_ was calculated in the same concentration range (0.0625–0.3125 mg/mL). The antiradical activity of TE and LE was statistically significantly lower than the Trolox used as a standard (*p* < 0.001: LE versus Trolox; 0.001 < *p* < 0.05: TE versus Trolox). Regarding the antioxidant power of the two extracts determined by the FRAP method, TE also showed stronger antiradical action than LE, as in case of the DPPH test. The FRAP values showed that there was a highly significant difference between LE and TE (*p* < 0.001). The results are in good agreement with the TPC values listed in Table 1, so that TE with a higher content of TPC (35.65 mg/g) exhibited greater antioxidant activity than LE (28.62 mg/g). The antioxidant activity of the Romanian grapes LE proved to be superior to those reported for different samples from Turkey [31,32] or lower than *V. vinifera* extracts from Serbia and Algeria [30,36].

Regarding grape tendrils, the data on the phenolic contents and the biological actions are very few or missing (derived from caffeic acids and antioxidant activity). Only Dawbaa et al. (2017) and Fraternale et al. (2011) obtained lower amounts of TPC and TFC [37,38].

The total phenolic content, as well as the antioxidant activity of the two ethanolic extracts, revealed quantitative differences between the studied samples, the TE being richer in polyphenolic compounds. Thus, the two secondary by-products of *V. vinifera* c.v. Fetească neagră could be a potential source of natural antioxidants used for designing nutraceutical/cosmetic products to improve human health.

### 3.2. Chromatographic Analysis of V. vinifera Fn Leaves and Tendrils Extracts

LC-MS/MS method led to the identification of ten polyphenolic compounds in *V. vinifera* Fn leaves and tendrils extracts through the comparison of retention times and the MS spectra with those of the standards. The identified compounds were further quantified based on their peak area and the calibration curve of their corresponding standards. The polyphenolic characterization was performed using an external standard method with 20 phenolic standards (nine phenolic acids and 11 flavonoids). Thus, no other phenolic substances could be determined in TE and LE.

As shown in Table 2, caftaric, protocatechuic, and gallic acids; catechin; epicatechin; hyperoside; isoquercitrin; rutin; quercitrin; and quercetol were determined in the two samples. Thus, the two samples showed a qualitatively identical phenolic profile, the differences being quantitative. The phenolic acids—gallic and protocatechuic acids—were found in higher quantities in tendrils (6.90 and 7.92 µg/g, respectively) compared to leaves (5.50 and 1.71 µg/g, respectively). Caftaric acid concentration was below the quantification limit of the analytical method (<0.02). The same seven flavonoids were found in the two samples. Catechin was 2.2 times higher in TE (51.72 µg/g) than in LE (34.31 µg/g), whereas epicatechin was found to be higher in leaves (7.10 µg/g), compared to tendrils (2.10 µg/g). The glycoside flavonoids: hyperoside, isoquercitrin, and quercitrin, as well as quercetol (flavonoid aglycone) were found in higher concentrations in LE (147.09, 903.49, 188.74, and 10.54 µg/g, respectively), compared to TE (127.39, 541.34, 100.87, and 8.89 µg/g, respectively). Only rutin was found in higher quantity in TE (617.21 µg/g), 1.6 times higher than in LE (385.63 µg/g). A one-way ANOVA test was used for the values in Table 2, and the statistical results (*p* < 0.001) supported the highly significant differences between the two extracts in terms of their polyphenolic composition. Some polyphenolic components, such as gallic acid, caftaric acid, catechin, rutin, and quercetin, have been quantified in leaf extracts by other authors [29,36], whereas for tendrils, few data were found in the literature. It can be concluded that *V*. *vinifera* leaves can be an important source of isoquercitrin, whereas tendrils are rich in rutin, two flavonoids with proven therapeutically potential (antioxidant, antimicrobial, anti-inflammatory, anticancer, etc.). Both leaves and tendrils could be components of a variety of nutraceutical, medicinal, pharmaceutical, and dermo-cosmetic products [39,40].

### 3.3. Cytocompatibility and Cytoprotective Effect of LE and TE against Nicotine-Induced Cytotoxicity

**Figure 1:**
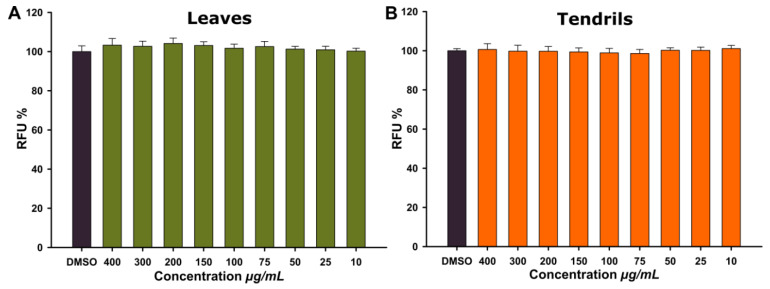
Cytocompatibility of the LE (**A**) and TE (**B**) observed using Alamar Blue assay on human gingival fibroblasts (HGF). The results are expressed as relative means ± standard deviations (six technical replicates for each of the three biological replicates) where the negative control (DMSO 0.2%) is 100%.

Cell morphology was assessed before and after the treatment with TE and LE by light microscope inspection. No morphological alterations in terms of spreading and cellular volume were noticed after the treatment with both extracts at all doses evaluated. The lack of toxicity was also supported by cell viability assessed using AB assay, with no sign of toxicity being observed in cells treated for 24 h with LE and TE at concentrations ranging from 10 to 400 µg/mL (Figure 1). In agreement with the current results, which indicated a high cytocompatibility of the extracts, Fraternale et al. reported that an aqueous extract from *V. vinifera* tendrils was not cytotoxic for human keratinocytes at a dose of up to 100 mg/mL [38]. Moreover, several other studies that evaluated the biological activities of aqueous or alcoholic extracts from different parts of *V. vinifera* indicated relatively low cytotoxicity on cultured cells, with the cancerous cell types displaying a higher sensitivity towards the cytotoxic effects of the extracts [41,42,43].

**Figure 2:**
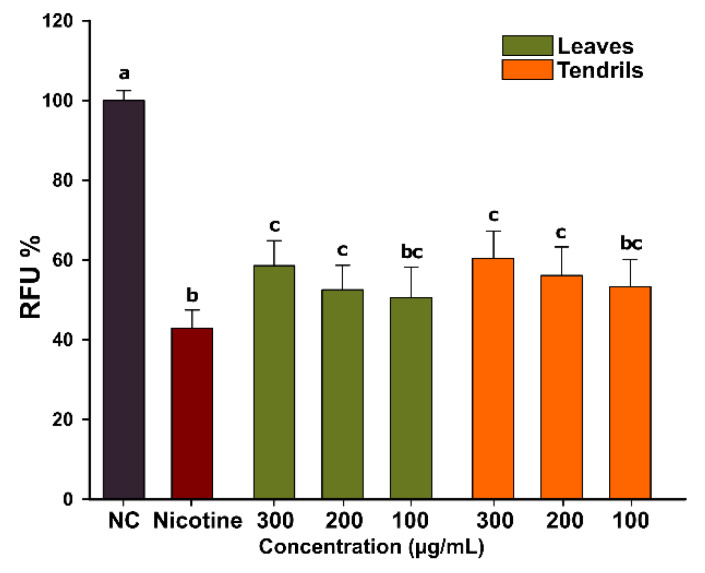
Cytoprotective effect of LE and TE against nicotine-induced cytotoxicity was analyzed after a 24 h post-exposure to three concentrations of the LE and TE in combination with 10 μM nicotine using AB assay. The values are expressed as mean ± standard deviation (SD) of three biological replicates. Different letters (a–c) represent the significant differences in mean cellular viability (ANOVA + Holm-Sidak post hoc test at *p* < 0.05 level of significance).

Cigarette smoking is a primary environmental risk factor for the initiation and/or progression of periodontal disease [44]. The effects of the whole tobacco smoke and of individual components, including nicotine, have been examined in numerous studies where an inflammatory and immune response were noticed [45]. Moreover, nicotine has been shown to have an additive detrimental effect in periodontitis, the combination with LPS, the main factor incriminated in periodontitis, increasing the expression of the metalloproteinases in osteoblasts [46], stimulating the formation and action of osteoclast-like cells [47], and increasing the expression of proinflammatory cytokines in fibroblastic cells [48]. Recently, natural compounds and plant extracts rich in polyphenolic and flavonoid content, capable of mitigating the host inflammatory response, have received considerable attention in the treatment of periodontitis [49]. In this regard, we evaluated the possible cytoprotective effects of LE and TE in HGF exposed to cytotoxic doses of nicotine. Exposure for 24 h to nicotine at a concentration of 10 µM decreased the cellular viability by approximately 60%, the cells displaying a decrease in the cellular volume and surface spreading. Co-exposure to the TE and LE had a cytoprotective effect, decreasing the nicotine cytotoxicity at the two highest tested doses (Figure 2). At the highest concentration tested of 300 µg/mL, both extracts decreased the nicotine toxicity by approximately 20%. No difference in potencies between the two extracts was observed as a two-way ANOVA with dose and type of extract as variables indicated that only the dose is influencing the cytoprotective response against nicotine. Similar to current results, Desjardins et al. reported that epigallocatechin-3-gallate (EGCG), an ester of epigallocatechin and gallic acid, had a protective effect at doses between 1 and 5 µg/mL in human gingival fibroblasts and oral epithelial cells exposed to toxic doses nicotine [8]. The authors hypothesized that the beneficial effects of EGCG are due to the proven antioxidant activity, as nicotine exerts its toxicity by increasing the ROS level and by depletion of cellular thiol content [11]. Moreover, EGCG has been shown to chelate transition metals that increase ROS levels by the Fenton reaction and to indirectly upregulate the expression of phase II antioxidant enzymes [50]. Numerous other compounds present in the TE and LE extracts, such as the caffeic acid derivatives, quercetin, and its esters, have also been shown to have a protective effect against nicotine induced cytotoxicity [9,10,51,52,53].

### 3.4. Antioxidant Potential

**Figure 3:**
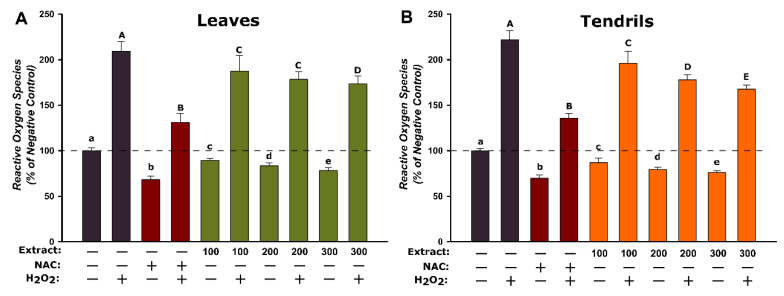
Antioxidant effect of the LE (**A**) and TE **(B**) evaluated using DCFH-DA assay on HGF cells. The cellular model was pre-exposed to LE and TE (100, 200, and 300 µg/mL) or NAC (20 mM) for 24 h, and further incubated with 50 µM DCFH-DA. The antioxidant effect was evaluated after 2 h on stimulated (250 µM H_2_O_2_) and un-stimulated conditions. The results are expressed as relative means ± standard deviations (six technical replicates for each of the three biological replicates) where the negative control (DMSO 0.2%) is 100%. Different letters (a–e refers to comparisons on non-stimulated conditions, whereas A–E refers to comparisons on stimulated conditions) represent significant differences (ANOVA + Holm-Sidak post hoc test at *p* < 0.05 level of significance).

Three concentrations of extracts (100, 200, and 300 µg/mL) were selected to evaluate the antioxidant effect. Exposure to the extracts alone led to a dose-dependent decrease of the basal oxidative status compared to the negative control. Similarly, N-acetylcysteine (NAC) treatment significantly reduced the quantity of ROS in non-stimulated condition (Figure 3). Both variables, extract type and dose, significantly influenced the basal oxidative status in HGF cells, the TE displaying a higher antioxidant capacity than LE. In stimulated conditions, following the H_2_O_2_ exposure, an increase of ROS partially inhibited by pretreatment with the TE, LE, and NAC was noticed. The higher antioxidant potential of TE noticed on non-stimulated conditions was confirmed on stimulated conditions, where pre-incubation with TE decreased the ROS in a dose-dependent manner. In the case of LE, a statistical decrease in the oxidative status was observed at all doses; however, the response was not dose-dependent (Figure 3). The results obtained indicate an antioxidant potential in in vitro cell cultures, and are consistent with other studies evaluating the antioxidant potential of different extracts from *V. vinifera* [54,55]. Based on the same assay as employed in the current study, Trindade et al. reported that an aqueous extract of *Vitis labrusca* cv. Isabella leaves possessed antioxidant properties at concentrations ranging between 0.5 and 5 µg/mL. The antioxidant potential of a grape skin extract was also reported in isolated mitochondria using the DCFH-DA assay [56]. In addition to the decrease in ROS, the aqueous extract of tendrils has been shown to increase in a dose- and time-dependent manner the reduced glutathione concentrations in cultured human keratinocytes, thus increasing the main cellular antioxidant defense mechanism [38]. In a recent article, Marabini et al. reported that the grapevine LE at a concentration of 100 µg/mL had a DNA-protective effect against UV-A and UV-B radiation in HaCaT cells by mitigating the induced oxidative stress [57]. The current findings are supported by the results of non-cellular assays, DPPH and FRAP, obtained within the present study, but also by other reports of the antioxidant potential of extracts from different parts of the *V. vinifera* in non-cellular assays such as FRAP, DPPH, and TEAC [58,59,60,61]. In addition to the above-mentioned assays, the antioxidant effects of *V. vinifera* extracts were also documented in animal and human studies. The majority of studies describe the antioxidant effects of grape (seeds, skin, and juice), which mitigates some of the alterations induced by the exposure of rats/mice to lead, CCl_4_, ethanol and γ-radiation [60,61,62,63]; however, in the case of the leaves and tendrils, two important by-products, the available data is scarce. Saadaoui et al. reported that the LE of two varieties of grapevine had a gastroprotective effect on ethanol-induced gastritis in rats. Moreover, the extracts prevented the decrease in the activity of several antioxidant enzymes and the increase in lipid peroxidation in the stomach tissue [64]. The beneficial effects of *V. vinifera* extracts were also documented in human studies. A randomized, double-blind, crossover clinical study using elite athletes reported that consumption of a grape extract standardized in flavanols permitted an improvement of the oxidative stress/antioxidant status balance during a competition period [65].

### 3.5. Anti-Inflammatory Potential

Due to the observed antioxidant potential and the composition of the extracts that included compounds with known anti-inflammatory potential, such as polyphenols, especially flavonoids (isoquercitrin and hyperoside), the capacity of the LE and TE to inhibit induced inflammation was evaluated. To test the effect of the LE and TE on IL-8, IL-6, and IL-1β release, HGF cells were challenged with pure lipopolysaccharide (LPS) from *Escherichia coli* (*E. coli*), which induce cellular inflammation. To exclude a possible additive/synergic toxicity of extract–LPS mixture, the viability of cells incubated with 100 ng/mL LPS and the three concentrations of each extracts that were previously selected was evaluated with no sign of toxicity being observed (data not shown).

**Figure 4:**
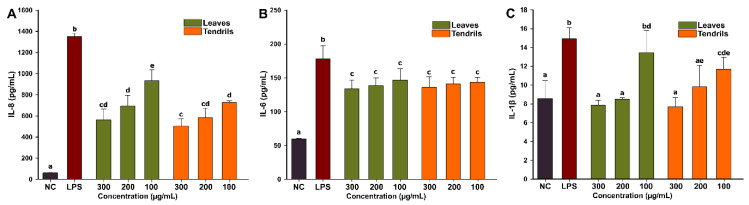
The extracellular release of proinflammatory cytokines IL-8 (**A**), IL- 6 (**B**), and IL-1β (**C**) was analyzed in cell-free supernatants by ELISA at 24 h post-exposure to three concentrations of the LE and TE in combination with 100 ng/mL LPS. The values are expressed as mean ± standard deviation (SD) of four biological replicates. Different letters (a–e) represent the significant differences in mean quantities of cytokines measured (ANOVA + Holm-Sidak post hoc test at *p* < 0.05 level of significance).

Exposure of cells to 100 ng/mL LPS induced a potent inflammatory response, the level of the inflammatory cytokines increasing by approximately 22-, 3-, and 1.7-fold for IL-8, IL-6, and IL-1β, respectively (Figure 4). Co-exposure to both extracts partially preempted the inflammatory response elicited by the LPS exposure, with all proinflammatory cytokine levels decreasing in the cellular supernatants. Except for the lowest concentration of LE, which did not reduce the level of IL-1β, all other conditions led to a statistical decrease in the concentrations of the evaluated cytokines. At the highest tested dose, LE decreased the levels of IL-8 and IL-6 by approximately 60% and 40%, respectively, whereas for IL-1β, the extract reduced the cytokine concentration to the basal level. Similarly, exposure to the TE decreased the levels of IL-8 and IL-6 by approximately 65% and 40%, respectively, whereas for IL-1β, a decrease to the basal level was observed. In case of IL-8 secretion, both the dose and the type of extract influenced the response, with TE displaying a higher anti-inflammatory potential than LE, whereas for IL-1β no differences between the extract potencies were noticed. Even though in case of IL-6, both extracts significantly reduced the amount of released cytokines, no relationship between the dose and the anti-inflammatory potential was established. Sangiovanni et al. reported that an aqueous extract from *V.* *vinifera* leaves prevented the increase in the IL-8 secretion in human epithelial gastric and intestinal cells challenged with TNF-α [66]. The exerted anti-inflammatory potential was due to a decrease in the nuclear translocation of NF-kB, a transcription factor that induces the expression of several proinflammatory cytokines, including IL-8 [67]. Interestingly, the above-mentioned authors evaluated the anti-inflammatory potential of the extract before and after an in vitro digestion step meant to mimic the human gastrointestinal digestion, with the results indicating that the digestion step is associated with a decrease in the anti-inflammatory potential, most probably due to the degradation of the bioactive components of the extract [66]. Similarly, Fraternale et al. reported that a hydroalcoholic extract of *V*. *vinifera* tendrils had anti-inflammatory potential and decreased the proinflammatory effects associated with LPS exposure in human endothelial cells and immune cells [7]. Another important by-product of winemaking highly abundant in vitamin E and polyphenolic compounds is represented by the grape seeds [68]. The unsaponifiable fraction isolated from grape seed oil has been shown to skew the monocyte plasticity towards the anti-inflammatory subtype and to inhibit the expression and secretion of TNF-α, IL-1β, and IL-6 in LPS-treated human primary monocytes [69]. The anti-inflammatory potential of the *V. vinifera* extracts is further supported by in vivo data [70,71].

Showing significant anti-inflammatory properties, bioactive compounds from natural sources have received an increased attention for the management of various inflammatory-related diseases. The epidemiological, clinical, and animal studies conducted so far indicate that polyphenols, the most abundant antioxidants in the human diet, are beneficial in the management of cardiovascular, neurodegenerative, and metabolic diseases [72,73]. In this context, several studies evaluated the beneficial effects of different polyphenols in complex mixtures such as natural extracts or as individual compounds in the treatment of periodontal disease [49]. Moreover, the use of natural compounds with a limited toxicity and with additional antimicrobial properties could be an alternative to the use of non-steroidal anti-inflammatory drugs (NSAIDs) as adjuvant therapy in the periodontal disease [74]. Most of the studies conducted so far on this topic evaluated the effects of extracts obtained from *Camellia sinensis* (green and black tea) and from cranberries [74]. To the best of our knowledge, we report for the first time the cytoprotective, antioxidant, and anti-inflammatory properties of TE and LE, and based on our results, their possible use in the management and treatment of periodontal disease. It should be emphasized that the above-mentioned beneficial effects should be viewed from a general perspective, as the relationship between antioxidant and anti-inflammatory effects is intricate and the observed effects are not entirely separated [75]. Polyphenolic compounds that were shown to activate the nuclear translocation of the ROS-sensitive Nrf2/Antioxidant Response Element pathway, and thus act as indirect oxidants by increasing the expression of antioxidant elements, have also been shown to possess anti-inflammatory properties. By activating the Nrf2 pathway, the nuclear translocation of the Nf-kB transcription factor, responsible for the synthesis of proinflammatory molecules, is inhibited, explaining the overlapping of the antioxidant and anti-inflammatory effects [76].

### 3.6. Antimicrobial Activity

Dental caries and periodontal disease, as the most prevalent oral diseases, are considered the main cause of tooth loss [77]. The etiology of dental caries is multifactorial involving the conversion of free sugars from food and drinks into acids by the microbial biofilm, thereby leading to the demineralization of the enamel surface. The most prominent factor is *Streptococcus mutants*, identified in more than 90% of the isolates associated with human caries [78]. Previous studies have shown that *Candida albicans* could enhance the adherence of *Streptococcus mutans* at the tooth surface, underlying the role of this commensal fungal species in caries development [79].

Unlike tooth decay, which is a relatively fast process caused by the long-term high intake of sugars, periodontal disease develops in several years. Periodontitis is mainly associated with *Porphyromonas gingivalis, Prevotella intermedia, Fusobacterium nucleatum, Tannerella forsythia, Treponema denticola*, *Aggregatibacter actinomycetemcomitans*, and *Enterococcus faecalis* [80].

Additionally, the presence of removable dental prosthesis induces an alteration of the oral microflora. Even some microorganisms that are uncommon in the oral microbiota, many of which being pathogenic, such as *Staphylococcus aureus*, *Streptococcus pneumoniae*, *Haemophilus influenzae*, *Haemophilus parainfluenzae*, *Escherichia coli*, *Klebsiella pneumoniae*, *Proteus mirabilis*, *Enterobacter cloacae*, and *Pseudomonas aeruginosa*, have been isolated from denture plaque. Therefore, dental prosthesis can represent a reservoir of pathogens microorganisms becoming potential sources of infections [81]. In this regard, we investigated the antimicrobial activity against seven strains involved in oral pathology. At the end of the incubation period at 37 °C, the diameters of inhibition zones (mm) were determined for the microbial strains tested. It was observed that in all strains the control sample (ethyl alcohol, 50%) showed inhibition 0, but the tested samples varied in size of inhibition diameter depending on the microbial strain tested (Table 3). For samples inoculated with bacteria *Streptococcus mutans* ATCC 25175, *Porphyromonas gingivalis* ATCC 33277, *Enterococcus faecalis* ATCC 29212, *Staphylococcus aureus* ATCC 25923, and *Escherichia coli* ATCC 25922 an inhibition of microbial growth was observed for all types of studied extracts applied in wells. LE presented a greater inhibition than TE, except for the strain *S. aureus* ATCC 25923, for which the TE presented the highest inhibition diameter.

For the bacterial strain *Porphyromonas gingivalis* ATCC 33277, inhibition was observed in both samples tested, a higher value of inhibition diameter being obtained in case of LE. In recent years, numerous studies demonstrated in vitro antimicrobial activity of *V*. *vinifera* extracts on bacterial strains involved in periodontitis, mainly due to the high concentration of flavonoids and stilbenes, responsible for the biological activity [82]. Thus, Furiga et al. demonstrated the bacteriostatic effect of grape seed extracts on two periodontopathogens, *Porphyromonas gingivalis* and *Fusobacterium nucleatum*, with high values for minimum inhibitory concentration [83].

In the case of other bacteria present in the bacterial biofilm, such as *Streptococcus mutans* ATCC 25175, the diameter of the inhibition zones was lower as compared to *Porphyromonas gingivalis.* The antibiofilm activity of grape seed extracts against *Streptococcus mutans* is well documented, but only few studies reported the antimicrobial activity of leaves and tendrils extracts. Thus, Yim et al. reported a significant antimicrobial activity against *Streptococcus mutans* of stilbenes and oligostilbenes from the leaves and stems of *Vitis amurensis* [84].

Our findings showed that both leaves and tendrils were effective against *Enterococcus faecalis* ATCC 29212 with a slight difference between the two samples. In an ex vivo study in human teeth, Checcin et al. reported a significant reduction in *Enterococcus faecalis* levels in root canals, without interfering with mechanical properties of dentine [85].

Of all the microbial strains evaluated in this study, for the bacterial strain *Staphylococcus aureus* ATCC 25923, the highest inhibition (14.5 mm) was observed for TE, whereas for LE, a diameter of the inhibition zone of 12 mm was observed. For the bacterial strain *Escherichia coli* ATCC 25922 inhibition was observed for both TE (10 mm) and LE (11.5 mm). In a previous study, Abed et al. showed that leaves extracts of *V. vinifera* were effective against *Staphylococcus aureus*, but ineffective against *Escherichia coli* [86]. In our study, TE exhibited the highest inhibition against bacterial strain *Staphylococcus aureus*, that may be correlated with the higher content of catechin, rutin, and protocatechuic acid found in TE as also reported by other authors [87,88,89].

For the bacterial strain *Klebsiella* sp. no inhibition was observed for any of the tested samples. We only observed the migration of the extract around the disk but without presenting an inhibition zone. For the fungal strain *Candida albicans* ATCC 10231 no inhibition was observed for any of the tested samples. Moreover, this strain developed only in the form of small colonies isolated from each other.

Despite the different phytochemical composition, TE and LE showed similar biologic activities with slight differences between the two extracts, which could be attributed to main components, but also to the synergic or antagonistic effects characteristic to phytocomplexes [90].

## 4. Conclusions

Winery industry by-products represent valuable and inexpensive sources of bioactive compounds. In this context, our study aimed to increase the knowledge regarding the composition and the possible utility in oral care products of the leaves and tendrils extracts from *V. vinifera*. The results obtained indicate that both matrices are rich sources of flavonoids, caffeic acid derivatives and polyphenols. As a result of the mentioned composition, both extracts displayed an antioxidant potential in the DPPH and FRAP assays. The antioxidant effect observed in the non-cellular assays was confirmed by the cellular DCFH-DA assay, both extracts decreasing the ROS levels on stimulated and non-stimulated conditions. In addition, both TE and LE demonstrated anti-inflammatory capabilities by mitigating the proinflammatory response induced by the LPS exposure of HGF cells. To the best of our knowledge, we report for the first time the cytoprotective, antioxidant, and anti-inflammatory properties of TE and LE, and based on our results, their possible use in the management and treatment of periodontal disease. The extracts had notable antimicrobial effects on several pathogenic bacteria from oral cavity, such as *Porphyromonas gingivalis*, *Enterococcus faecalis*, *Streptococcus mutans*, and *Staphylococcus aureus*, which may be associated with oral pathology. The above-mentioned results encourage us to move forward to the next stage of research by formulating oral cavity care products using tendrils and leaves extracts.

## Figures and Tables

**Table 1:** Total phenolic contents and antioxidant activity of *V*. *vinifera* Fn LE and TE.

Samples	TFC (mg RE/g)	Caffeic Acid Derivatives (mg CAE/g)	TPC (mg GAE/g)	IC_50_ (DPPH) (mg/mL)	FRAP (µmol Te/mL)
TE	14.21 ± 0.20 ^a^	4.14 ± 0.07 ^a^	35.65 ± 0.33 ^a^	0.155 ± 0.04 ^b,c^	10.60 ± 0.39 ^a^
LE	16.75 ± 0.12	6.39 ± 1.11	28.62 ± 0.24	0.248 ± 0.01 ^a^	6.29 ± 0.20
Trolox	-	-	-	0.011 ± 0.00	-

Legend: Fn: Feteasca neagra; TFC: total flavonoid content; TPC: Total polyphenols content; CAE: caffeic acid equivalents GAE: gallic acid equivalents; RE: rutin equivalents; Te: Trolox equivalents. Values (mean ± SD, *n* = 3) marked with uncapitalized letters showed statistical differences: ^a^ *p* < 0.001 (LE versus TE; LE versus Trolox); ^b^ *p* < 0.05 (LE versus TE); ^c^ 0.001 < *p* < 0.05 (TE versus Trolox).

**Table 2:** Polyphenols identified in *V. vinifera* LE and TE by liquid chromatography coupled with mass spectrometry in tandem (LC-MS/MS) (µg/g plant material).

Polyphenolic Compounds	*m/z* Value	t_R_ ± SD (min)	LE (µg/g)	TE (µg/g)
Gallic acid	169	1.50 ± 0.01	5.50 ± 0.05	6.90 ± 0.11 ^a^
Protocatechuic acid	153	2.80 ± 0.01	1.71 ± 0.02	7.92 ± 0.08 ^a^
Caftaric acid	311	3.54 ± 0.05	< 0.02	<0.02
Catechin	289	6.00 ± 0.03	23.31 ± 0.36	51.72 ± 1.06 ^a^
Epicatechin	289	9.00 ± 0.01	7.10 ± 0.09	2.60 ± 0.09 ^a^
Hyperoside	463	18.60 ± 0.12	147.09 ± 1.79	127.39 ± 2.61 ^a^
Isoquercitrin	463	19.60 ± 0.10	903.49 ± 6.51	541.34 ± 6.44 ^a^
Rutin	609	20.20 ± 0.15	385.63 ± 3.36	617.21 ± 6.79 ^a^
Quercitrin	447	23.64 ± 0.13	188.74 ± 2.26	100.87 ± 2.13 ^a^
Quercetin	301	26.80 ± 0.15	10.54 ± 0.24	8.89 ± 0.11 ^a^

Note: Each value is the mean ± SD of three independent measurements. ^a^ *p* < < 0.001 (LE versus TE).

**Table 3:** The diameter of inhibition zone (mm).

Bacterial/Fungal Strain	Inhibition Diameter for the Tested Samples (mm) ± SD		
	C	1	2
*Porphyromonas gingivalis* ATCC 33277	0 ± 0.00	10 ± 0.00	13 ± 1.41
*Enterococcus faecalis* ATCC 29212	0 ± 0.00	10 ± 0.00	12 ± 1.41
*Streptococcus mutans* ATCC 25175	0 ± 0.00	10 ± 0.00	11.5 ± 0.71
*Staphylococcus aureus* ATCC 25923	0 ± 0.00	14.5 ± 0.71	12 ± 1.41
*Escherichia coli* ATCC 25922	0 ± 0.00	10 ± 1.41	11.5 ± 0.71
*Klebsiella* sp.	0 ± 0.00	0 ± 0.00	0 ± 0.00
*Candida albicans* ATCC 10231	0 ± 0.00	0 ± 0.00	0 ± 0.00

Legend: 1 = TE; 2 = LE; C—ethyl alcohol/H_2_O 50/50 (*v/v*).
